# Evaluation of inflammatory parameters in patients with hepatic hydatid disease

**DOI:** 10.1080/07853890.2021.1966084

**Published:** 2021-08-18

**Authors:** Zhijia Fan, Yao Hu, Li Wang, Haoqin Jiang, Dandan Li, Hui Zhao, Zhicheng Wang

**Affiliations:** a Department of Laboratory Medicine, Huashan Hospital, Fudan University, Shanghai, China; b Department of Laboratory Medicine, First Affiliated Hospital of Xinjiang Medical University, Urumqi, China

**Keywords:** Echinococcosis, platelet, prognostic nutritional index, gamma-glutamyl transpeptidase to platelet ratio, alkaline phosphatase to platelet ratio

## Abstract

**Background:**

To the best of our knowledge, the association of inflammatory parameters with hepatic hydatid disease (HD) has not been investigated in a single study. We aimed to evaluate the potential value of inflammatory indices in this disorder.

**Methods:**

The retrospective study including 114 patients was performed from January 2016 to November 2019. Clinical characteristics and laboratory data for all participants were collected and analysed. The levels of inflammatory parameters were compared in the patient and control group, the predictive value of these inflammatory parameters was assessed by the logistic regression analysis and receiver operating characteristic curve, and differences between pre- and post-surgical operations were compared by pair tests.

**Results:**

Significantly higher levels of platelet distribution width (PDW), eosinophil percentage (EOS %), neutrophil to lymphocyte ratio (NLR), gamma-glutamyl transpeptidase to platelet ratio (GPR) and alkaline phosphatase to platelet ratio (APPR) and lower levels of platelet (PLT) and prognostic nutritional index (PNI) were observed in patients than in controls. Multivariate analyses showed that hydatid could induce the abnormal levels of these parameters, of which APPR and PNI had more obvious changes as compared to other parameters. The levels of PDW and APPR significantly decreased after surgical treatment.

**Conclusions:**

Inflammatory parameters closely associated with the hepatic HD could be used in the evaluation of treatment as assistant indexes.

**KEY MESSAGE**
- Hydatid disease (HD) seriously endangers public health and economic development.
- Inflammatory parameters that are readily available and acceptable in routine clinical practice could be closely associated with HD.
- Inflammatory parameters could be used in the evaluation of disease development by combing with histological and radiological results in future studies.

## Introduction

Hydatid disease (HD) is a zoonotic parasitic disease caused by the adult or larval stages of Echinococcus cestodes. In recent years, HD has become a common and near-cosmopolitan disease that seriously endangers public health and brings economic burden [1]. China, as one of the countries representing high risks, is estimated to account for 91% of the global incidence [2,3]. Besides, western China is the key area reporting the highest prevalence of echinococcosis [4]. The control and even elimination of echinococcosis are still very serious due to the increasing substantially morbidity and mortality [5].

Humans accidentally ingest the parasite eggs and the larvae are subsequently liberated into the intestine. They could usually reach the liver through the portal system and also disperse to other organs by invading the bloodstream. The liver and lungs are the most commonly infected organs [6–8]. However, owing to the lack of clinical symptoms in the early-stage, early diagnosis and treatment of hepatic HD are difficult [4]. Hepatic HD is mainly diagnosed by imaging results and serological tests currently [4]. The growth and proliferation of the hydatid are directly related to an immune-inflammatory reaction by infiltration of inflammatory cells [9] and thus, some haematologic inflammatory factors which are simple and accessible in routine laboratory tests for use as auxiliary markers may be considered. Other than that some typical blood parameters in routine clinical tests, including leukocytes and thrombocytes representing markers of inflammation, some calculated parameters derived by combing blood count with liver function markers, such as prognostic nutritional index (PNI) [10], gamma-glutamyl transpeptidase to platelet ratio (GPR) [11] and alkaline phosphatase to platelet ratio (APPR) [12], have also attracted broad attention due to their inflammatory role.

However, these inflammatory parameters have not previously been evaluated in hepatic HD and little is known about the correlation between them. Our study aimed to retrospectively compare the levels of these parameters between patients and healthy individuals, simultaneously observe changes in these parameters after surgical treatment.

## Materials and methods

### Study population and study design

In this retrospective study, a total of 177 patients diagnosed with hepatic HD from January 2016 to November 2019 were enrolled and the normal group was recruited from the centre of Health Examination. The Medical Ethics Committee of First Affiliated Hospital, Xinjiang Medical University approved the protocol (approval no. K201906-06), in accordance with the Declaration of Helsinki.

The inclusion criteria: (1) the definitive diagnosis of hepatic HD based on clinical evaluation, radiological findings and laboratory tests; (2) age 18 years or older; (3) complete information of electronic medical records. The exclusion criteria: (1) no surgical therapy of removing hydatid; (2) incomplete postoperative laboratory data. Finally, the data of 114 patients were analysed.

### Data collection and definitions

The clinical characteristics of patients were obtained from the electronic medical records: age, sex, type of infection, comorbidities, haematological parameters and liver function tests. The complete blood count parameters including red cell count (RBC), white cell count (WBC) and platelet (PLT) count were analysed by the impedance method on a Sysmex XN-2000 hematology analyzer (Sysmex Corporation, Kobe, Japan). The blood liver function parameters including serum gamma-glutamyl transpeptidase (GGT), alkaline phosphatase (ALP), alanine aminotransferase (ALT), aspartate aminotransferase (AST), albumin values were measured by the dry-chemistry method on a VITROS 5600 automated chemistry analyzer (Ortho Clinical Diagnostics, Raritan, NJ). They were used for the calculation of inflammatory parameters. The PLR was defined as PLT to lymphocyte ratio; NLR was defined as neutrophil to lymphocyte ratio; GPR was defined as GGT to PLT ratio; APPR was defined as ALP to PLT ratio; ALPR was defined as ALT to PLT ratio; APRI was defined as AST to PLT ratio; PNI was calculated as serum albumin level (g/L)+5 × total lymphocyte count (×10^9^/L). Postoperative haematological parameters within a month after the operation were also collected for analyses in all patients.

### Statistical analysis

Statistical analyses were done using SPSS statistics 22 (IBM SPSS, Inc., Armonk, NY) and GraphPad Prism 6 (GraphPad Software, La Jolla, CA). Continuous data were presented as mean ± standard deviation or median (interquartile range), which were compared by the *t*-test or the Mann–Whitney *U* test, as appropriate. Univariate and multivariate logistic regression analyses, after correction for potential confounding factors such as age, gender and ethnic minorities, were applied to evaluate the risk factors of hepatic HD. Odds ratios (ORs) with 95% confidence intervals (CIs) were calculated for the estimated association. The relationships among the inflammatory indices were analysed using Spearman’s correlation and multiple testing was correlated using Bonferroni’s analysis. A paired *t*-test or a paired Wilcoxon’s rank test was performed to compare changes of parameter levels between baseline and after surgery. A *p* value <.05 was considered statistically significant.

## Results

### Patient characteristics

**Figure 1.**
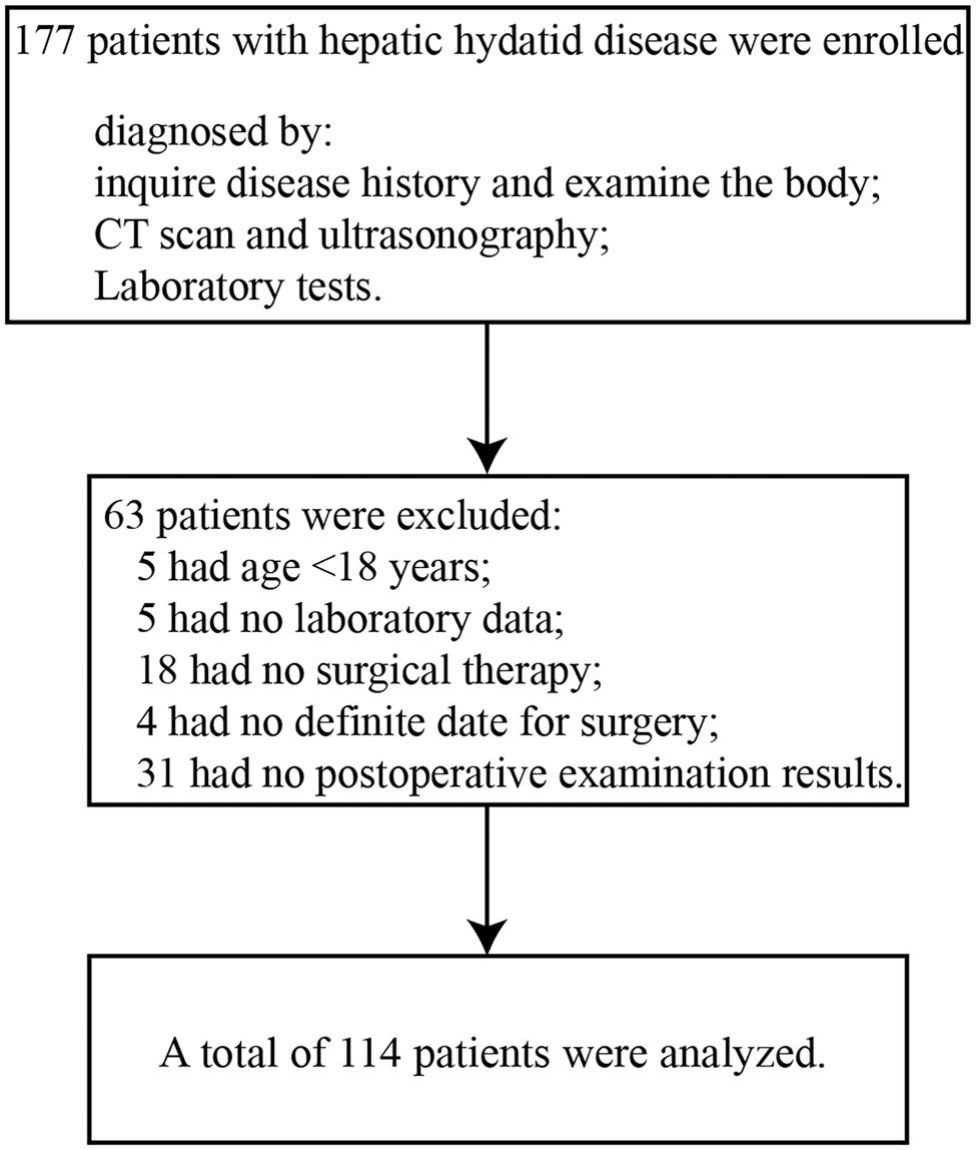
Flowchart of exclusion criteria.

A total of 114 patients with hepatic HD were analysed in this study based on the inclusion and exclusion criteria (Figure 1). The baseline clinical characteristics and laboratory data are listed in Table 1. Seventy-six patients did not find that they had hepatic HD until through medical examination. Most patients (69%) were accompanied by chronic cholecystitis.

**Table 1.** Clinical and haematological characteristics of the study groups.

Variable	Patient group (*N* = 114)	Control group (*N* = 114)	*p* Value
Demographics			
Sex, *n* (%)			.232
Male	57 (50%)	66 (58%)	
Female	57 (50%)	48 (42%)	
Ages (years), median (IQR)	45 (36, 53)	47 (36, 54)	.637
Liver biochemistry			
Serum albumin (g/L), mean ± SD	39.4 ± 5.8	45.8 ± 2.9	<.0001
Serum globulin (g/L), mean ± SD	33.2 ± 8.3	28.2 ± 3.5	<.0001
ALT (U/L), median (IQR)	22.9 (16.9, 38.7)	21.0 (15.6, 35.5)	.374
AST (U/L), median (IQR)	20.5 (17.0, 28.5)	22.5 (18.6, 29.1)	.086
GGT (U/L), median (IQR)	38.1 (21.0, 91.5)	25.7 (16.9, 45.5)	.0007
ALP (U/L), median (IQR)	90.6 (67.3, 148.0)	62.3 (54.1, 73.1)	<.0001
Laboratory results			
RBC count (×10^9^/L), mean ± SD	4.64 ± 0.60	4.99 ± 0.54	<.0001
PLT count (×10^9^/L), mean ± SD	241 ± 64.1	267 ± 65.1	.003
PDW (fL), mean ± SD	14.1 ± 2.98	12.5 ± 2.13	<.0001
MPV (fL), mean ± SD	10.5 ± 1.11	10.5 ± 0.98	.955
WBC count (×10^9^/L), mean ± SD	6.56 ± 2.13	6.62 ± 1.55	.821
NEU count (×10^9^/L), median (IQR)	3.74 (2.73, 4.41)	3.59 (2.90, 4.43)	.898
NEU %, median (IQR)	57.7 (50.5, 65.9)	56.0 (51.8, 61.6)	.260
LYM count (×10^9^/L), median (IQR)	1.76 (1.38, 2.33)	2.14 (1.65, 2.48)	.0004
LYM %, median (IQR)	28.6 (23.4, 36.7)	32.6 (28.4, 37.0)	.006
EOS count (×10^9^/L), median (IQR)	0.20 (0.10, 0.35)	0.13 (0.06, 0.21)	<.0001
EOS %, median (IQR)	3.05 (1.70, 5.93)	2.05 (1.00, 3.00)	<.0001
Inflammatory indices			
NLR, median (IQR)	1.99 (1.41, 2.66)	1.72 (1.40, 2.13)	.047
PLR, median (IQR)	130 (105, 164)	129 (101, 156)	.443
PNI, mean ± SD	48.8 ± 7.66	56.5 ± 4.57	<.0001
ALPR, median (IQR)	0.09 (0.07, 0.15)	0.09 (0.05, 0.16)	.095
APRI, median (IQR)	0.091 (0.068, 0.133)	0.088 (0.065, 0.125)	.549
GPR, median (IQR)	0.16 (0.10, 0.37)	0.10 (0.06, 0.18)	<.0001
APPR, median (IQR)	0.39 (0.30, 0.61)	0.24 (0.19, 0.30)	<.0001
Detection by medical examination, *n* (%)			
Yes	76 (67%)		
No	38 (33%)		
Type of disease, *n* (%)			
New	98 (86%)		
Recurrence	16 (14%)		
Echinococcosis infection, *n* (%)			
Cystic	89 (78%)		
Alveolar	25 (22%)		
Chronic cholecystitis, *n* (%)			
Yes	79 (69%)		
No	35 (31%)		

ALT: alanine aminotransferase; AST: aspartate aminotransferase; GGT: gamma-glutamyl transpeptidase; ALP: alkaline phosphatase; RBC: red blood cell; PLT: platelet; PDW: platelet distribution width; MPV: mean platelet volume; WBC: white blood cell; NEU: neutrophil; LYM: lymphocyte; EOS: eosinophil; NLR: neutrophil to lymphocyte ratio; PLR: platelet to lymphocyte ratio; PNI: prognostic nutritional index; ALPR: alanine aminotransferase to platelet ratio ; APRI: aspartate aminotransferase to platelet ratio index; GPR: gamma-glutamyl transpeptidase to platelet ratio; APPR: alkaline phosphatase to platelet ratio.

The results showed that serum albumin, GGT and ALP had obvious differences between the control and patient group, while no significant differences in ALT and AST were observed. A significant increase in GGT and ALP suggested the impairment of the patient’s hepatobiliary system.

Some laboratory results, such as RBC, WBC and PLT count, were lower in patients, while platelet distribution width (PDW) and eosinophil percentage (EOS %) were higher in patients than in healthy individuals. But the levels of mean platelet volume (MPV) were similar between the two groups.

We found significant inflammatory response in patients, as evidenced by the altered levels of inflammatory indices, the patient group had higher levels of NLR (*p*=.047), GPR (*p*<.0001) and APPR (*p*<.0001) and lower levels of PNI (*p*<.0001) with statistical significance as compared to those in the control group.

### Association of inflammatory indices with disease

Table 2 summarizes the results of binomial logistic regression analyses about inflammatory parameters. These inflammatory indices were all significantly correlated with the occurrence of hepatic HD both in the univariate logistic regression analysis and in the multivariate analysis adjusted for confounding factors.

**Table 2.** Univariate and multivariate analyses to identify risk factors associated with hydatid disease.

Variable	Univariate analysis		Multivariate analysis^a^	
	OR (95%CI)	*p* Value	OR (95%CI)	*p* Value
PLT (×10^9^/L)	0.994 (0.990–0.998)	.004	0.993 (0.988–0.997)	.001
PDW (fL)	1.279 (1.148–1.426)	<.0001	1.313 (1.172–1.470)	<.0001
EOS %	1.242 (1.108–1.391)	.0002	1.257 (1.117–1.414)	.0001
NLR	1.527 (1.130–2.062)	.006	1.545 (1.140–2.093)	.005
PNI	0.795 (0.744–0.850)	<.0001	0.768 (0.712–0,830)	<.0001
APPR	2.164 (1.645–2.847)	<.0001	2.344 (1.749–3.142)	<.0001
GPR	1.260 (1.087–1.462)	.002	1.299 (1.101–1.533)	.002

PLT: platelet; PDW: platelet distribution width; EOS: eosinophil; NLR: neutrophil to lymphocyte ratio; PNI: prognostic nutritional index; APPR: alkaline phosphatase to platelet ratio; GPR: gamma-glutamyl transpeptidase to platelet ratio.

^a^ Adjusted for age, gender and ethnic minorities.

Here, we showed that the development of HD was negatively associated with PLT (OR, 0.993; *p*=.001) and PNI (OR, 0.768; *p*<.0001), while was positively associated with PDW (OR, 1.313; *p*<.0001), EOS % (OR, 1.257; *p*=.0001), NLR (OR, 1.545; *p*=.005), APPR (OR, 2.344; *p*<.0001) and GPR (OR, 1.299; *p*=.002) (Table 2).

### Correlation analyses among inflammatory indices

As shown in Table 3, we analysed the correlations among inflammatory indices in the patient cohort. APPR was significantly related to all other parameters. In particular, after multiple testing, a stronger positive correlation was calculated between APPR and GPR (*r* = 0.657, *p*<.0001). GPR was positively associated with EOS % (*r* = 0.301, *p*=.001) and negatively associated with PNI (*r*=–0.326, *p*=.0004). PNI was also found to be correlated significantly with other indices, except for no correlation with PLT. PLT could interfere with the analysis of PDW as there existed a significantly negative correlation between them (*r*=–0.356, *p*=.0001).

**Table 3.** Correlation among inflammatory parameters of patients.

Variable	PLT	PDW	EOS %	NLR	PNI	APPR
GPR						
*r*	–0.073	–0.188	0.301	0.149	–0.326	0.657
*p*	.440	.045^a^	.001^b^	.113	.0004^b^	<.0001^b^
APPR						
*r*	–0.230	–0.196	0.267	0.217	–0.429	
*p*	.014^a^	.037^a^	.004^b^	.020^a^	<.0001^b^	
PNI						
*r*	0.062	0.360	–0.211	–0.467		
*p*	.513	<.0001^b^	.024^a^	<.0001^b^		
NLR						
*r*	–0.026	–0.039	–0.023			
*p*	.785	.682	.811			
EOS %						
*r*	0.091	–0.141				
*p*	.337	.135				
PDW						
*r*	–0.356					
*p*	.0001^b^					

PLT: platelet; PDW: platelet distribution width; EOS: eosinophil; NLR: neutrophil to lymphocyte ratio; PNI: prognostic nutritional index; GPR: gamma-glutamyl transpeptidase to platelet ratio; APPR: alkaline phosphatase to platelet ratio; *r*: correlation coefficient.

^a^ *p*<.05.

^b^ *p*<.01.

### Comparison of inflammatory indices between pre- and post-operation

The levels of inflammatory parameters before and after surgery are also compared in Table 4. PDW and MPV values were lower in the postoperative period compared to those before therapy. ALP markedly decreased accompanied by the significant reduction of APPR. No significant differences in PLT and EOS % were observed around treatment, while PNI still kept lower after surgery (Table 4).

**Table 4.** Comparison of haematological parameters of patients before and after surgical treatment.

Variable	Preoperative period	Postoperative period	*p* Value
PLT count (×10^9^/L), mean ± SD	241 ± 64.1	242 ± 68.6	.878
PDW (fL), mean ± SD	14.1 ± 2.98	11.9 ± 2.32	<.0001
MPV (fL), mean ± SD	10.5 ± 1.11	10.2 ± 1.04	.0006
EOS %, median (IQR)	3.05 (1.70, 5.93)	3.40 (0.98, 7.20)	.321
PNI, mean ± SD	48.8 ± 7.66	39.9 ± 5.38	<.0001
ALP (U/L), median (IQR)	90.6 (67.3, 148.0)	77.9 (63.0, 121.0)	<.0001
APPR, median (IQR)	0.39 (0.30, 0.61)	0.37 (0.27, 0.50)	<.0001
GGT (U/L), median (IQR)	38.1 (21.0, 91.5)	45.3 (22.1, 80.7)	.010
GPR, median (IQR)	0.16 (0.10, 0.37)	0.18 (0.11, 0.36)	.028

PLT: platelet; PDW: platelet distribution width; MPV: mean platelet volume; EOS: eosinophil; PNI: prognostic nutritional index; ALP: alkaline phosphatase; APPR: alkaline phosphatase to platelet ratio; GGT: gamma-glutamyl transpeptidase; GPR: gamma-glutamyl transpeptidase to platelet ratio.

## Discussion

Hepatic HD may be undetected for many years because it is often asymptomatic in the early stage unless enlarging lesions and compressing organs lead to complications, and HD usually presents in a single organ [13–15]. In the present study, 67% of patients discovered hydatid accidentally on a routine medical examination and only 18% of patients had two or more organ involvement that supported the findings above. Our study suggested the significantly abnormal expression of inflammatory parameters in patients with HD. PDW and APPR could be used as follow-up markers in patients after surgical resection.

Aside from their crucial role in the modulation of haemostasis, inflammation and immune response, PLTs have cytotoxic effects against parasites and are capable of killing them [16,17]. We observed significantly lower PLT levels in patients compared with those in healthy individuals. Reduced thrombopoietin production due to the derangement of liver function and increased PLT consumption during the process against hydatid may cause low PLT expression in patients. Besides, PDW and MPV representing PLT activation were also studied to be associated with HD [17]. In line with previous studies, our results showed that PDW was higher in patients and had a significant reduction after surgery. Additionally, we detected a significantly negative correlation between the PLT and PDW. Likewise, MPV decreased markedly after treatment although no significant difference was observed in the patient and control group. In consequence, these findings suggested a close association between abnormal number and function of PLTs and hepatic HD.

Eosinophilia, a more specific change for parasitic infection, was significantly associated with complicated lesions [18,19]. Elevation of eosinophil count and percentage in patients was observed in our study. Considerable numbers of eosinophils could be recruited into the inflammatory site during the stage of initiation and even proliferation of hydatid [20,21].

Recent research found that a connection between the liver and biliary tract was one of the most common lesions involvement of hydatid [22]. Most patients had chronic cholecystitis suggesting the harmful impact of the hydatid cysts on the biliary system. Preoperative ALP and GGT, rather than ALT or AST, were determined as potential markers for cyst-biliary communication in hepatic HD [23,24]. Our results well highlighted the predictive value of ALP and GGT that a significant increase in both parameters was observed in patients.

Meanwhile, the study tried to connect blood count with liver function indexes for new inflammatory parameters, such as APPR, GPR and PNI for the potential role in the management of hepatic HD. We found higher levels of APPR and GPR and lower PNI levels in patients. Besides, they could be combined for evaluation due to the strong correlations among the three indices. Of note, a significant reduction in APPR after surgical therapy was also found. Based on these results, it was suggested that APPR may be useful in the diagnostic value of hepatic HD as well as in follow-up after treatment.

Low PNI not only indicated increased inflammation but showed poor nutrition status. Propagation and dissemination of the parasites accompanied by liver tissue damage could decrease albumin production. Significantly decreased PNI was discovered in the postoperative period. It was probably because albumin still decreased as a negative acute-phase protein and the function of damaged hepatocytes was not yet recovered in the short-term follow-up after surgery. Further studies would be needed to perform the long-term follow-up for the evaluation of liver function by inflammatory indices mentioned above.

Although the development of HD occurs insidiously and slowly, it is mostly lifelong that threatens public health and imposes high economic losses on patients. Vaccination research against echinococcosis including live vaccines, DNA vaccines and recombinant protein vaccines has attracted much attention to reducing the transmission level [25]. Moreover, recent research demonstrated that a multi-antigenic recombinant vaccine successfully interrupted the infection transmission cycle by controlling the pathogen in both intermediate and definitive hosts, simultaneously [26]. Vaccination would be an effective tool for the prevention of the disease.

There are several limitations in this study. First, this single-centre retrospective study may have inherent biases due to missing data. Second, the clinical data of hepatic lesions such as the size and number of cysts were not recorded in detail. Larger sample size and multiple-centre studies that extending postoperative follow-up time would clarify the value of inflammatory indices in hepatic HD.

In summary, our results presented a remarkable correlation between hepatic HD and inflammatory parameters. A significant increase in PDW, EOS, NLR, GPR and APPR and a decrease in PLT and PNI were found in patients, particularly elevated PDW and APPR, which gradually returned to normal levels after surgical treatment. Intrahepatic dissemination of the parasites could cause a body-wide inflammatory reaction and influence these parameters’ expression levels. In future research, these parameters may be conducive as follow-up markers in patients with HD who undergo surgical resection and the associations of inflammatory parameters with histological and radiological results will need to be further explored.

## Ethical approval

The Medical Ethics Committee of First Affiliated Hospital, Xinjiang Medical University approved the protocol (approval no. K201906-06), in accordance with the Declaration of Helsinki.

## Data Availability

The data that support the findings of this study are available from the corresponding author, upon reasonable request.
